# Preferred Supramolecular Organization and Dimer Interfaces of Opioid Receptors from Simulated Self-Association

**DOI:** 10.1371/journal.pcbi.1004148

**Published:** 2015-03-30

**Authors:** Davide Provasi, Mustafa Burak Boz, Jennifer M. Johnston, Marta Filizola

**Affiliations:** Department of Structural and Chemical Biology, Icahn School of Medicine at Mount Sinai, New York, New York, United States of America; Baltimore, United States of America

## Abstract

Substantial evidence in support of the formation of opioid receptor (OR) di-/oligomers suggests previously unknown mechanisms used by these proteins to exert their biological functions. In an attempt to guide experimental assessment of the identity of the minimal signaling unit for ORs, we conducted extensive coarse-grained (CG) molecular dynamics (MD) simulations of different combinations of the three major OR subtypes, i.e., μ-OR, δ-OR, and κ-OR, in an explicit lipid bilayer. Specifically, we ran multiple, independent MD simulations of each homomeric μ-OR/μ-OR, δ-OR/δ-OR, and κ-OR/κ-OR complex, as well as two of the most studied heteromeric complexes, i.e., δ-OR/μ-OR and δ-OR/κ-OR, to derive the preferred supramolecular organization and dimer interfaces of ORs in a cell membrane model. These simulations yielded over 250 microseconds of accumulated data, which correspond to approximately 1 millisecond of effective simulated dynamics according to established scaling factors of the CG model we employed. Analysis of these data indicates similar preferred supramolecular organization and dimer interfaces of ORs across the different receptor subtypes, but also important differences in the kinetics of receptor association at specific dimer interfaces. We also investigated the kinetic properties of interfacial lipids, and explored their possible role in modulating the rate of receptor association and in promoting the formation of filiform aggregates, thus supporting a distinctive role of the membrane in OR oligomerization and, possibly, signaling.

## Author Summary

Opioid receptors associate with each other in the plasma membrane, following a mechanism that has been implicated in either beneficial or adverse effects, depending on the environment and the interacting partners. Whether or not the different opioid receptor subtypes share a similar propensity to form di-/oligomers and specific receptor-receptor interactions is still unclear on the basis of the published data. This information, however, is necessary to predict stabilizing or de-stabilizing mutations and to design experiments to clarify the role of oligomerization in opioid receptor function. The inferences provided by the extensive molecular dynamics simulations reported herein constitute a first step in this direction.

## Introduction

Experimental evidence accumulated over almost two decades supports the ability of all three major opioid receptor (OR) subtypes (μ-OR, δ-OR, and κ-OR) to form homomeric and heteromeric complexes, a feature that is common to several other G Protein-Coupled Receptors (GPCRs) (see [1] for a recent review). In particular, heteromerization between μ-OR and δ-OR and between δ-OR and κ-OR has been suggested to expand the repertoire of OR signaling by modulating ligand binding, receptor signaling, and/or trafficking properties [2]. This modulation has a direct translational relevance in view of the putative role that OR heteromers play in opioid-induced adverse effects (e.g., the development of analgesic tolerance [3,4]), and in the observed potentiation of morphine-induced analgesia by δ-OR-selective antagonists [5]. Thus, targeting OR oligomers directly may lead to novel drugs with potentially greater selectivity and reduced side effects compared to molecules targeting individual receptors.

While a few ligands have already been proposed to target OR heteromers (e.g., see [6–11]), it remains unclear how they bind and activate these receptor complexes. To begin addressing these questions and eventually use these and other molecules as tools to elucidate the physiological relevance of OR oligomers, ligand-bound models of these receptor complexes are highly desirable. However, building such models is not feasible in the absence of reliable information about their interface of dimerization/oligomerization in a physiologically relevant environment. The recent X-ray crystal structures of the μ-OR [12] and κ-OR [13] have suggested specific receptor-receptor interactions involving transmembrane (TM) helices TM5 and TM6 or TM1, TM2, and helix 8 (H8). Although these interfaces are thermodynamically stable according to our recent free-energy calculations of μ-OR and κ-OR homo-dimers in an explicit lipid-water environment [14], the possibility cannot be ruled out that other receptor-receptor interactions are also feasible in the cell membrane, and perhaps more kinetically favorable than those inferred from crystallography.

Here, we carried out extensive, unbiased coarse-grained (CG) molecular dynamics (MD) simulations of freely diffusing ORs in an explicit lipid-water environment to evaluate differences and similarities in the supramolecular organization and preferred dimeric interfaces of all three major receptor subtypes.

## Results and Discussion

A summary of all the MD simulations we carried out on CG molecular models based on the crystallographic structures of inactive δ-OR [15], μ-OR [12], and κ-OR [13] is provided in S1 Table. Specifically, to enhance the statistical significance of our description of the dimerization process, we ran five independent simulations for each of the homomeric (i.e., μ-OR/μ-OR, δ-OR/ δ-OR, and κ-OR/κ-OR) and the most studied heteromeric (i.e., δ-OR/μ-OR and δ-OR/κ-OR) complexes of ORs, starting from randomized initial orientations of CG models of sixteen individual receptors (8+8 in the case of heteromers) in an explicit CG palmitoyl-oleoyl-phosphatidyl-choline (POPC)/10% cholesterol bilayer. Together, these simulations yielded over 250 μs of accumulated data, which correspond to approximately 1 ms of effective simulated dynamics, according to the established (×4) scaling factor of the CG MARTINI model [16–18] we employed.

### Preferred Supramolecular Organization of ORs

**Fig 1:**
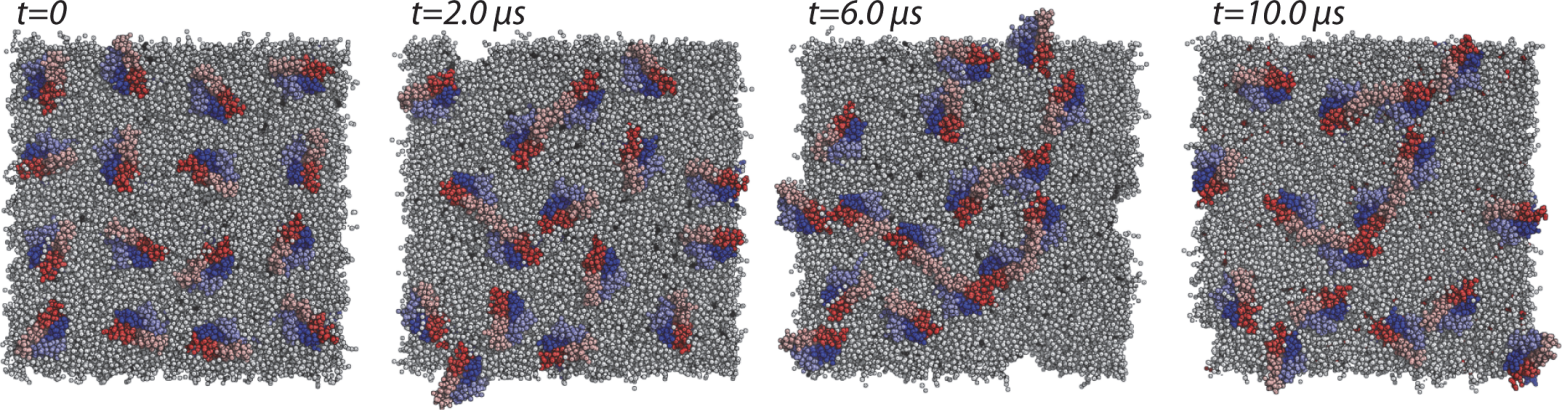
An example of OR setup at different simulation times. Location of the 16 simulated receptor molecules in one of the five runs executed for the κ-OR system (run #1), at different simulation times, (specifically: 0, 2, 6, and 10 μs)

All the twenty-five different simulations we carried out showed multiple association events between ORs initially located far apart and moving freely in the lipid bilayer. As an example, Fig. 1. shows the location of each of the 16 receptor molecules in one of the five simulations executed on the κ-OR system (run #1) at different simulation times (specifically 0, 2, 6, and 10 μs).

While the formation of several receptor-receptor complexes could be observed during simulation of the μ-OR/μ-OR, δ-OR/ δ-OR, κ-OR/κ-OR, δ-OR/μ-OR, and δ-OR/κ-OR systems, each individual protomer in the complex seldom shared more than two interfaces, thus favoring filiform rather than branched or compact high-order arrangements (see simulation snapshots at 6 and 10 μs in Fig. 1, as well as S1–S5 Movies). Notably, linear arrays of receptors were observed in early atomic force microscopy images of the prototypic GPCR rhodopsin in native membranes [19], and were further supported by early CG simulations [20]. Below, we speculate that the formation of chains of receptors may be influenced by lipid dynamics (see the Dynamic Behavior of Lipid Molecules section for a rationale).

Importantly, once formed, no receptor complex dissociated over the maximal simulated time of 10 μs, but rather only minor interface rearrangements were observed. This observation is in line with experimental estimates of dimer lifetime (a few seconds) obtained from recent single-molecule imaging studies of different, individually labeled, GPCRs in living cells [21]. Since dissociation is not observed in the simulations reported here, a direct calculation of free-energies is precluded. However, analysis of the executed twenty-five simulations instills confidence in that they might have captured the majority of fastest-forming dimer interfaces of ORs.

### Preferred Homo-dimer and Hetero-dimer Interfaces of ORs

The five different simulation trajectories obtained for each OR system were pooled together to derive statistically meaningful information about the various dimer interfaces that formed during simulation. Since each protomer structure was kept fairly rigid by elastic network forces during the simulations (see Materials and Methods for details), no allosteric communication between protomers or inter-dependence between dimer interfaces were expected. Interfaces were defined based on the minimal number of residues on each receptor TM helix within a certain distance cutoff from each other, and were clustered based on the similarity between inter-protomer contact maps (see details of the analysis under Materials and Methods).

Tables 1 and 2 report the preferred OR homo-dimer and hetero-dimer interfaces, as derived from Bayesian analysis of the pooled trajectories. The following interesting observations can be made on the basis of this analysis. First of all, not all possible combinations of TMs were found to be involved in dimer interfaces during the simulated timescale of 10 μs. The only interfaces that formed in all studied homo- and heteromeric systems are: TM1,2,H8/TM1,2,H8, TM1,2/TM4,5 (also TM4,5/TM1,2 for hetero-dimers), and TM1,2/TM5,6 (also TM5,6/TM1,2 for hetero-dimers). Other interfaces, such as TM4,5/TM4,5, TM4,5/TM5,6, TM5/TM5, and TM5/TM1,2 did not form in at least one of the five studied μ-OR/μ-OR, δ-OR/ δ-OR, κ-OR/κ-OR, δ-OR/μ-OR, and δ-OR/κ-OR systems. In particular, the TM4,5/TM4,5 interface, which has been suggested to constitute a possible GPCR dimer interface by various experimental assays (reviewed in [22]), only formed during simulation of the δ-OR/δ-OR system, and with a lower frequency with respect to other interfaces formed by δ-OR homomers. Notably, neither TM3 nor TM7 were ever found to be involved in a dimer interface, whether formed by the same or different receptor subtypes.

**Table 1:** Fraction of the observed homo-dimeric interfaces during five independent MD simulations of the μ-OR/μ-OR, δ-OR/δ-OR, and κ-OR/κ-OR systems.

Interface	δ-OR/δ-OR		κ-OR/κ-OR		μ-OR/μ-OR	
TM1,2,H8/TM1,2,H8	17.4	(9,27.4)	26.0	(16.8,36.2)	7.8	(2.2,16.6)
TM1,2/TM4,5	41.3	(29.4,53.3)	27.2	(18.1,37.7)	9.8	(3.3,18.8)
TM1,2/TM5,6	31.7	(21,43.5)	22.1	(13.7,31.8)	37.3	(24.7,50.9)
TM4,5/TM4,5	1.6	(0,5.7)	―	―	―	―
TM4,5/TM5,6	7.9	(2.6,15.7)	1.3	(0,4.7)	1.9	(0,7)
TM5/TM5	―	―	23.3	(14.6,33.3)	43.2	(30.3,56.8)

Best estimate of the fraction of each homo-dimeric arrangement and its (2.5%,97.5%) confidence intervals (in parenthesis) are calculated as reported in the Methods section.

**Table 2:** Fraction of the observed hetero-dimeric interfaces during five independent MD simulations of the μ-OR/δ-OR and δ-OR/κ-OR systems.

Interface	δ-OR/κ-OR		δ-OR/μ-OR	
TM1,2,H8/TM1,2,H8	19.10	(6.7,36.8)	17.70	(4.1,33.8)
TM1,2/TM4,5	15.40	(4.2,31.4)	5.80	(0.2,20.4)
TM1,2/TM5,6	15.50	(4.4,31.1)	17.60	(3.9,38.4)
TM4,5/TM1,2	18.90	(6.6,35.2)	11.80	(1.5,30.4)
TM4,5/TM5,6	19.40	(7,36.5)	―	―
TM5/TM1,2	―	―	11.80	(1.4,30.4)
TM5/TM5	―	―	29.30	(10.8,52.2)
TM5,6/TM1,2	11.60	(2.6,26.1)	5.80	(0.2,19.7)

Best estimate of the fraction of each hetero-dimeric arrangement and its (2.5%,97.5%) confidence intervals (in parenthesis) are calculated as reported in the Methods section.

Another important observation from our study is that TM helices appear with different frequencies at a dimer interface depending on the OR system. Specifically, the TM1,2 helices appear most frequently at the observed dimer interfaces of δ-OR and κ-OR, followed by TM4,5 and TM5,6, with the latter helix pair being more involved in a dimer interface in μ-OR compared to δ-OR and κ-OR. However, it is noted that the calculated confidence intervals for frequencies of the specific interfaces are quite broad and overlapping, and therefore the estimated differences between the three OR subtypes may not be as relevant as they appear to be.

### Comparison with Putative Dimer Interfaces of GPCRs Inferred from Crystallography

Several interfaces observed in the simulations reported here are structurally similar to some of the putative dimer interfaces inferred from recent GPCR crystal structures (see S2 Table for a list of currently available GPCR crystal structures showing parallel receptor arrangements). To allow a quantitative comparison, we calculated the minimum Cα root mean square deviation (RMSD) distance between members of the cluster of dimeric complexes that formed during the simulations and each crystal structure listed in S2 Table. The cases where this distance resulted to be less than 10 Å are reported in S3 Table for homo-dimers and in S4 Table for hetero-dimers.

**Fig 2:**
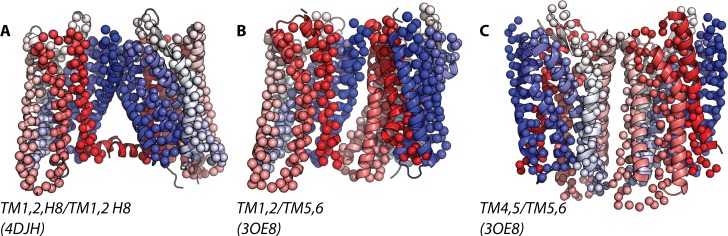
Superposition of the highly frequent homo-dimer configurations of δ-OR onto the closest available crystal structures of parallel receptors. Specifically, these are: TM1,2,H8/TM1,2,H8, TM1,2/TM5,6, and TM4,5/TM5,6, in panels A (RMSD of 3.57 Å from 4DJH), B (RMSD of 6.48 Å from 3OE8), and C (RMSD of 6.62 Å from 3OE8), respectively.

**Fig 3:**
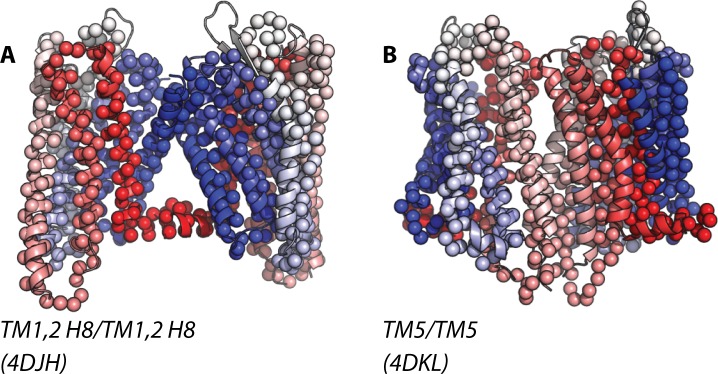
Superposition of the highly frequent homo-dimer configurations of κ-OR onto the closest available crystal structures of parallel receptors. Specifically, these are: TM1,2,H8/TM1,2,H8 and TM5/TM5 in panels A (RMSD of 4.13 Å from 4DJH) and B (RMSD of 8.56 Å from 3DKL), respectively.

**Fig 4:**
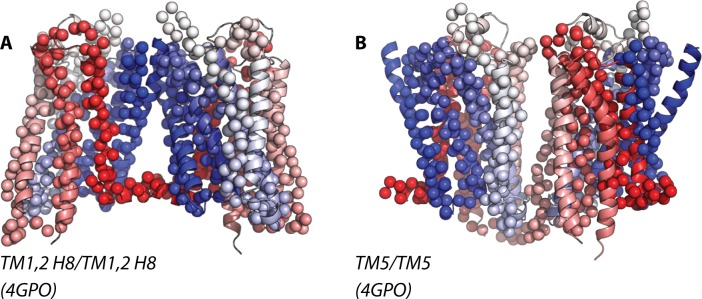
Superposition of the highly frequent homo-dimer configurations of μ-OR onto the closest available crystal structures of parallel receptors. Specifically, these are: TM1,2,H8/TM1,2,H8 and TM5/TM5 in panels A (RMSD of 5.92 Å from 4GPO) and B (RMSD of 8.82 Å from 4GPO), respectively.

The calculated RMSD values of S3 and S4 Tables suggest that the dimer interface from simulations that is closest to one inferred from crystal structures is the TM1,2,H8/TM1,2,H8 interface. In particular, δ-OR and κ-OR form TM1,2,H8/TM1,2,H8 interfaces in both homo- and hetero-dimers that are very close (RMSDs below 4.3 Å) to that seen in the crystal structure of κ-OR (4DJH [13]). The closest crystal structure to the TM1,2,H8/TM1,2,H8 interface that forms during μ-OR simulations is not the one inferred by the μ-OR crystal structure (4DKL [12]), but rather the one suggested by a β1-adrenergic receptor (B1AR) crystal structure (4GPO [23]). Figs. 2A, 3A, and 4A show, as an example, the TM1,2,H8/TM1,2,H8 homo-dimer interfaces formed during simulation of δ-OR, κ-OR, and μ-OR, respectively, overlapped onto the closest crystal structures, i.e., 4DJH or 4GPO.

The relatively small RMSD values listed in S3 Table, indicate that the simulations of the δ-OR system also reproduced both symmetric and asymmetric dimer interfaces inferred from CXCR4 crystal structures [24] (see S2 Table for details) with reasonable accuracy. Specifically, the interface herein termed TM1,2/TM5,6 deviated only 6.48 Å from the asymmetric interface revealed by 3OE8 (after overlapping the dimer from simulation with chains A and B of 3OE8) whereas the interface herein called TM4,5/TM5,6 deviated only 6.66 Å from an interface inferred from 3ODU (after overlapping the dimer from simulation with chains A and B of 3ODU) and 6.62 Å from an interface inferred from 3OE8 (after overlapping the dimer from simulation with chains B and C of 3OE8). Fig. 2B and 2C show structural overlaps of the TM1,2/TM5,6 and TM4,5/TM5,6 interfaces of δ-OR dimers with 3OE8.

Larger RMSD values were obtained for the identified TM5/TM5 interface in both κ-OR and μ-OR simulations (see S3 and S4 Tables) after comparison with the available GPCR crystal structures of interacting parallel receptors that are listed in S2 Table. Specifically, in κ-OR, this interface is 8.56 Å apart from the putative TM5,6-TM5,6 dimer interface inferred from the μ-OR crystal structure (after overlapping the dimer from simulation with chain A of 4DKL and its periodic image). Notably, μ-OR simulations did not produce dimeric arrangements that were close enough to the crystallographic TM5,6-TM5,6 interface of μ-OR, in spite of it being thermodynamically stable as we recently demonstrated through free-energy calculations [14]. The closest structure to the identified TM5/TM5 in μ-OR simulations was the interface termed TM4,5/TM4,5 in the B1AR crystal structure corresponding to PDB code 4GPO (RMSD of 8.82 Å, after overlapping the formed dimer with chain A of 4GPO and its periodic image). While the calculated RMSD of the identified TM5/TM5 interfaces of κ-OR and μ-OR homo- and hetero-dimers with respect to available crystal structures appear to be quite large, visual inspection of these overlaps (Figs. 3B and 4B for κ-OR and μ-OR, respectively) shows that the slight rotation of one protomer needed to match the two configurations would not dramatically change the nature of those interfaces. Thus, we speculate that the reason why the simulations reported herein are unable to reproduce the crystallographic TM5,6-TM5,6 interface of μ-OR is that this interface may need longer times to form than its slight modifications (more on this below).

### Insights into the Kinetics of OR Dimerization

To understand whether there are interfaces that are kinetically favored over others, i.e are fast forming in an explicit membrane environment, we calculated interface-specific dimerization rates (k_on_) for all simulated OR systems by fitting a Poisson model to the association instances observed during simulation. The results are reported in Tables 3 and 4 for the simulated homo- and hetero-dimers of ORs, respectively. The fastest forming homo-dimer interfaces are: TM1,2/TM4,5 and TM1,2/TM5,6 for δ-OR/δ-OR, TM1,2,H8/TM1,2,H8 and TM1,2/TM4,5 for κ-OR/κ-OR, and TM5/TM5 for μ-OR/μ-OR. Notably, TM1,2,H8/TM1,2,H8, TM4,5/TM1,2, and TM4,5/TM5,6 are the fastest interfaces for the δ-OR/κ-OR hetero-dimer, whereas the TM5/TM5 interface is the fastest forming for the δ-OR/μ-OR hetero-dimer. This observation further stresses the higher propensity for κ-OR to have the TM1,2 helices involved in a fast-forming dimer interface. In contrast, TM5 is more likely to be involved in a fast-forming dimer interface when one of the receptor partners is μ-OR.

**Table 3:** Association rates of OR homo-dimers formed during five independent MD simulations of the μ-OR/μ-OR, κ-OR/κ-OR, δ-OR/ δ-OR, δ-OR/μ-OR and δ-OR/κ-OR systems.

Interface	δ-OR		κ-OR		μ-OR	
TM1,2,H8/TM1,2,H8	8.4	(3.9,14)	20.5	(12.3,30.3)	4.0	(1,9)
TM1,2/TM4,5	20.1	(13.1,28.5)	21.5	(13.3,31.6)	5.1	(1.5,10.5)
TM1,2/TM5,6	15.3	(9.4,22.7)	17.3	(10.2,26.5)	19.4	(11.7,29.5)
TM4,5/TM4,5	0.7	(0,2.9)	―	―	―	―
TM4,5/TM5,6	3.9	(1.2,7.9)	1.0	(0,3.9)	1.0	(0,3.9)
TM5/TM5	―	―	18.5	(10.7,28.3)	22.7	(13.9,33.3)

Estimated k_on_ rates (in μm^2^/s) along with (2.5%, 97.5%) confidence intervals.

**Table 4:** Association rates of OR hetero-dimers formed during five independent MD simulations of the δ-OR/μ-OR and δ-OR/κ-OR systems.

Interface	δ-OR/κ-OR		δ-OR/μ-OR	
TM1,2,H8/TM1,2,H8	15.43	(4.9,31.5)	9.10	(1.9,22.1)
TM1,2/TM4,5	12.35	(3.2,26.2)	3.09	(0.2,11.4)
TM1,2/TM5,6	12.35	(3.2,27)	9.26	(1.9,22.2)
TM4,5/TM1,2	15.43	(4.9,30.9)	6.17	(0.8,17.4)
TM4,5/TM5,6	15.43	(4.9,30.9)	―	―
TM5/TM1,2	―	―	6.17	(0.6,17.1)
TM5/TM5	―	―	15.28	(4.9,31.5)
TM5,6/TM1,2	9.26	(1.9,22.2)	3.09	(0.2,11.6)

Estimated k_on_ rates (in μm^2^/s) along with (2.5,97.5) confidence intervals.

### Dynamic Behavior of Lipid Molecules

Analysis of the dynamic behavior of the POPC (herein referred to as ‘lipid’) and cholesterol molecules during the simulations reported here reveals considerable dynamical heterogeneity, in that regions of high mobility appear to be surrounded by slower molecules. Such a behavior, which is typical of glass-forming fluids and supercooled liquids [25], and was also suggested for membrane proteins (e.g., see [26–28]), has also been recently reported for water at the interface of globular proteins [29]. We speculate that this dynamical heterogeneity also controls membrane diffusion and viscosity near OR dimer interfaces, and plays an important role in modulating the rate of receptor association and the structure of the complex.

Visualization of the twenty-five simulation trajectories reported herein showed a clear variation in the dynamic behavior of lipid molecules during receptor dimerization. As seen in S1–S5 Movies, which are provided as representative simulation trajectories of the δ-OR/δ-OR, κ-OR/κ-OR, μ-OR/μ-OR, δ-OR/κ-OR, and δ-OR/μ-OR systems, respectively, the density of slow lipids becomes higher (dark blue in S1–S5 Movies) as protomers approach each other, suggesting that slow lipids may interfere with dimer formation at specific interfaces. To quantitatively investigate the role of lipid molecules in regulating OR dimerization, we calculated exchange and persistence time (t_X_ and t_P_, respectively) distributions of the lipids at different positions relative to isolated receptors. These two quantities characterize the diffusion properties (D) of the lipids and the effective viscosity (η) of the membrane around the protein, respectively (see details in the Methods section).

Typical observed average exchange times 〈t_X_〉 of lipids in the bulk membrane, i.e. away from the receptors, are ~10 ns (see S1 Fig.), corresponding to a lipid diffusion coefficient D≈d^2^/〈t_X_〉 = 10^−6^ cm^2^/s, (or D≈2.5×10^−7^ cm^2^/s, when accounting for the effective time scaling for the CG force field we used). In the simulations reported here, the average exchange time of lipids increases up to 〈t_X_〉≈40–50 ns at specific regions near the OR surface, giving D≈2.5×10^−7^ cm^2^/s (or, effectively, D≈6.2×10^−8^ cm^2^/s). Notably, similar values of lipid diffusion constants have recently been reported in the literature [26,27] for comparable CG force fields, and a similar behavior was implied.

**Fig 5:**
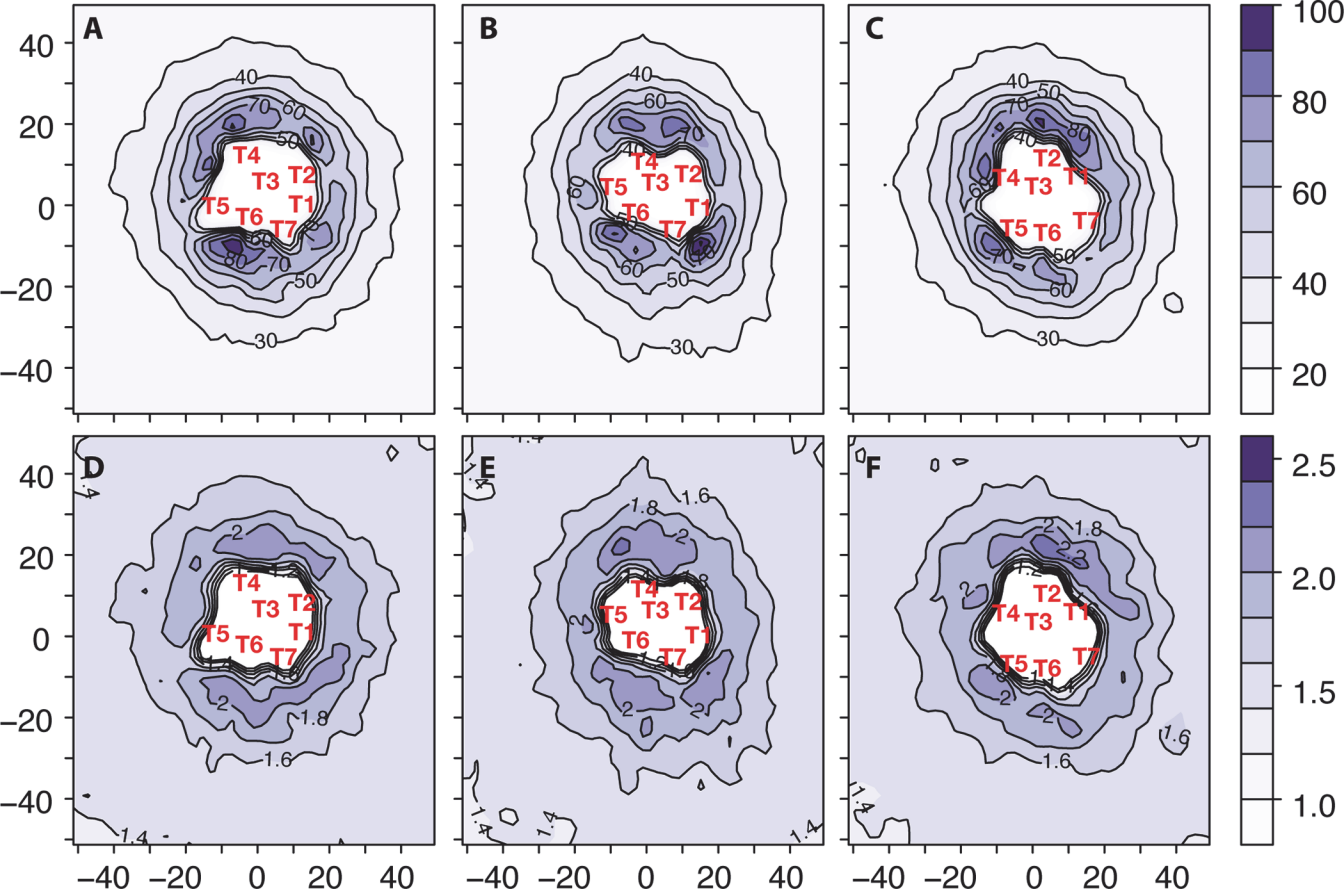
Persistence times and persistence-to-exchange time ratios of lipid molecules derived from the OR homo-dimer simulations. Specifically, persistence times of lipid molecules calculated for δ-OR/δ-OR, κ-OR/κ-OR, and μ-OR/μ-OR are reported, in ns, in panels A, B, and C, respectively. Persistence-to-exchange time ratios of lipids around isolated δ-OR, κ-OR, and μ-OR are shown in panels D, E, and F, respectively. Approximate locations of the centers of mass of the seven transmembrane helices (TM1-TM7) are indicated with red labels.

In the bulk membrane, the equivalence between the exchange and persistence times implies an inverse relationship between viscosity and diffusion coefficient in homogeneous systems, known as the Stokes-Einstein relation. The presence of dynamical heterogeneity, with slow lipids close to the protein surface, corresponds to a breakdown of the Stokes-Einstein relation for lipid dynamics in this region. In other words, correlated lipid motion leads to an increased membrane effective viscosity, decoupled from the diffusional motion of the lipids, so that the inverse relationship between the diffusion coefficient and viscosity (ηD ∝ constant), is no longer homogeneously valid. Analysis of the lipid dynamics around isolated ORs shows longer average persistence times 〈t_P_〉 (see Fig. 5, panels A, B, and C for δ-OR, κ-OR, and μ-OR, respectively) at specific locations of the protein surface, up to 100 ns. In general, persistence times increase more than exchange times 〈t_X_〉, so that the ratio 〈t_P_〉/〈t_X_〉 is usually larger than 1 (see Fig. 5, panels D, E, and F for δ-OR, κ-OR, and μ-OR, respectively). Usually, lipid molecules in the region of helices TM1,2 display the shortest persistence times during simulation compared to the region of helices TM4,5 and TM5,6. This is interesting in view of the observed prevalence of interfaces that involve helices TM1,2 and the corresponding fastest on-rates, compared to the generally less frequent participation of TM4,5 and TM5,6 in dimeric interfaces of ORs. Based on this observation, it is tempting to speculate that this local effect on the membrane viscosity (i.e., 〈t_P_〉/〈t_X_〉>1) and the presence of long-lived lipid microstates with long persistence times near the surface may delay the formation of specific interfaces, making them kinetically disfavored with respect to others.

The viscosity of the environment has a well-established, direct effect on the kinetics of biological processes as indicated by the expression of the rate constant in the widely applied Kramers’ framework [30]:

$$k≃\frac{m\omega^{†}\omega }{2\pi \eta }\exp\left(-\frac{G^{†}}{k_{\text{B}}T}\right)$$

where *m* is the effective mass associated with the order parameter used to describe the biological process, G^†^ is the free-energy of the transition state, ω and ω^†^ are the curvatures of the free-energy profile at the bottom and top of the barrier, and η is the viscosity. According to this expression, the higher viscosity observed in regions of the environment with long persistence times results in slower rates, and therefore slower kinetics of the process.

To quantitatively assess the relationship between regions of slow lipid dynamics around OR protomers and reduced kinetic rates, we calculated the position-dependent translational diffusion coefficients (D_P_) of μ-OR protomers at the identified homo-dimeric interfaces using a Bayesian inference approach described in the literature [31]. According to these calculations, the diffusion coefficients of μ-OR at homo-dimeric interfaces displaying slower on-rates (i.e., TM1,2,H8/TM1,2,H8, TM1,2/TM4,5, and especially TM4,5/TM5,6) are significantly reduced when the two protomers are within distances of a few nanometers. As reported in S5 Table, average diffusion rates of ~5×10^−7^ cm^2^/s estimated when the protomers are far apart (d>50 Å) are reduced to ~1–2×10^−7^ cm^2^/s as the inter-protomer distance reaches values between 40 and 50 Å. At shorter distances (d<40 Å), the diffusion rates decreased to less than 10^−7^ cm^2^/s for the slow-forming interfaces, but did not change much for the fast-forming interfaces (i.e, TM1,2/TM5,6 and TM5/TM5). This observation provides a possible explanation why the TM5,6/TM5,6 interface seen in the μ-OR crystal structure did not form during the 10 μs simulations, notwithstanding its thermodynamical stability [14]. As mentioned before, the two protomers of the TM5/TM5 dimer identified by simulations need to rotate to obtain the crystallographic TM5,6/TM5,6 configuration. We estimated the free-energy associated with such a rotation using the results of steered MD simulations, and assuming no conformational changes occurring within the individual protomers based on the CG model we employed. These results, which are reported in S4 Fig., are consistent with a high free-energy barrier (~10 kcal/mol) between the identified TM5/TM5 and the crystallographic TM5,6/TM5,6 dimers, further supporting the hypothesis that longer time scales are needed for the latter to form.

As also evident when viewing the S1–S5 Movies, lipid regions of length scales of a few nanometers between approaching receptors prior to dimer formation can also become locally trapped/restricted in motion (so-called “jammed” regions). While a complete kinetic analysis of the dimerization process that includes lipid dynamics cannot be achieved using the data reported herein, the simulations suggest a distinctive role for long-lived lipid microstructures as they appear to decrease the dimerization on-rate at specific interfaces, and kinetically select specific dimeric arrangements among all different possibilities.

**Fig 6:**
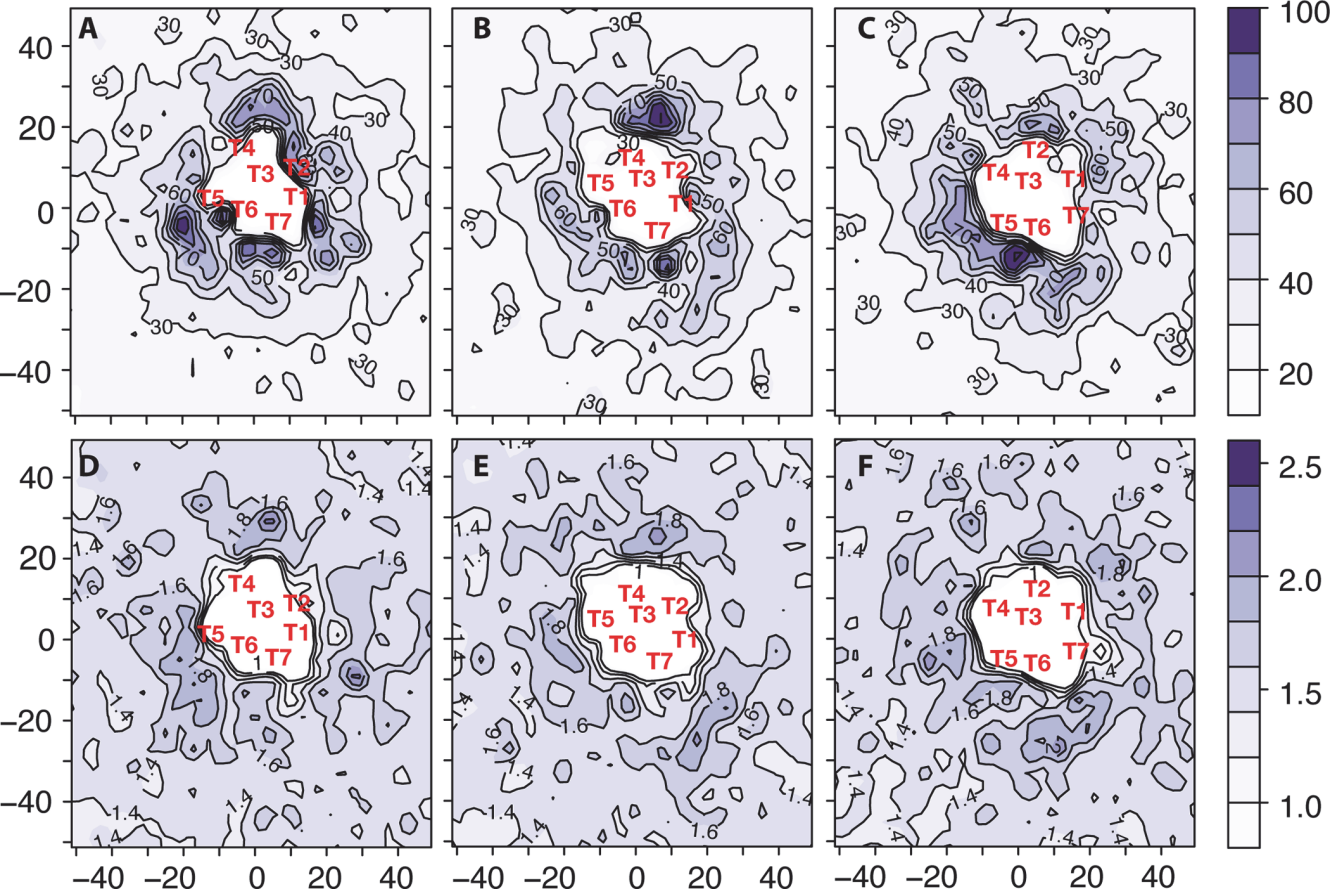
Persistence times and persistence-to-exchange time ratios of cholesterol molecules derived from the OR homo-dimer simulations. Specifically, persistence times of cholesterol calculated for δ-OR/δ-OR, κ-OR/κ-OR, and μ-OR/μ-OR are reported, in ns, in panels A, B, and C, respectively. Persistence-to-exchange time ratios of cholesterol around isolated δ-OR, κ-OR, and μ-OR are shown in panels D, E, and F, respectively. Approximate locations of the centers of mass of the seven transmembrane helices (TM1-TM7) are indicated with red labels.

We also investigated the dynamics of cholesterol molecules by calculating their persistence and exchange times around isolated δ-OR, κ-OR, and μ-OR, using the same strategy employed for the study of the lipid molecules. Average values of cholesterol exchange times are reported in S2 Fig., panels A-C, for δ-OR, κ-OR, and μ-OR, respectively, whereas average persistence times and persistence-to-exchange ratios of cholesterol molecules surrounding isolated δ-OR, κ-OR, and μ-OR are reported in Fig. 6A-C and 6D-F, respectively. Notably, regions with long cholesterol persistence times appear to be generally co-localized with regions with strong lipid molecule persistence, suggesting that both cholesterol-protein interactions and cholesterol-lipid interactions contribute to the kinetic selection of specific dimer interfaces.

Preferred cholesterol interacting sites at the surface of GPCR molecules have been reported in some of the published crystal structures. For instance, a cholesterol binding pocket was identified in a groove characterized by highly conserved residues (so-called “consensus-motif” residues) between the intracellular ends of helices TM2 and TM4 in two B2AR crystal structures, i.e., the carazolol-bound 2RH1 [32] and the timolol-bound 3D4S [33]. Cholesterol molecules were also observed in the ultra-high resolution crystal structure of the A2A adenosine receptor corresponding to PDB code 4EIY [34]. While the “consensus motif” residues identified in B2AR are conserved in A2A, no cholesterol was observed at the intracellular end of helices TM2 and TM4. In contrast, three cholesterol molecules were found at the extracellular sides of TM2,3, as well as TM5,6 and TM6,7. While no cholesterol molecules were resolved in the κ-OR or δ-OR crystal structures, electron density was attributed to a cholesterol molecule in the μ-OR crystal structure (4DKL), at the same location between TM6 and TM7 as seen in the A2A crystal structure 4EIY. Notably, the aforementioned “consensus motif” residues on TM2 (Y2.41, S2.45) are not conserved in members of the opioid receptor family. Moreover, the calculated high persistence time of cholesterol close to the extracellular ends of helices TM6 and TM7 from simulations of all three OR subtypes (see Fig. 6.) is consistent with the location of cholesterol molecules found in the ultra-high-resolution adenosine A2A crystal structure 4EIY and in the μ-OR crystal structure 4DKL. Although the palmitoylation site C3.55 at the intracellular end of TM3 was proposed to constitute part of a cholesterol preferred binding site in the groove lining the intracellular region between TM4 and TM5 [35], this region does not show increased persistence times of cholesterol molecules in the simulations reported here.

**Fig 7:**
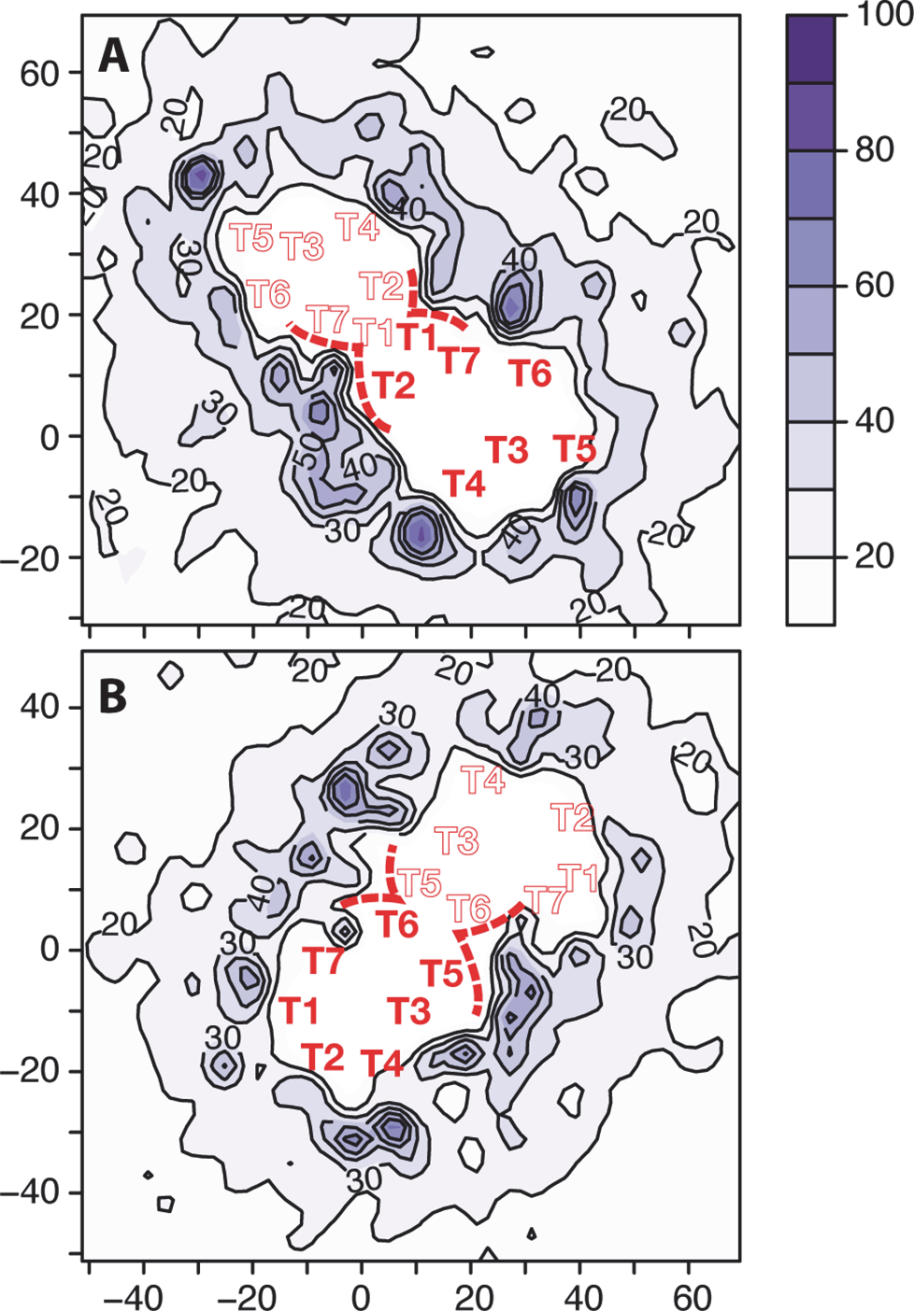
Persistence times of lipid molecules around μ-OR crystallographic interfaces using data from previously published simulations. Panels A and B show the persistence times of lipid molecules that are adjacent to the simulated TM1,2,H8/TM1,2,H8 and TM5,6/TM5,6 crystal dimers, respectively. Approximate locations of the centers of mass of the seven transmembrane helices (TM1-TM7) are indicated with solid and outlined red labels for each protomer, respectively. The lipid-exposed region of the protein surface adjacent to the interface is schematically highlighted with a red dashed line.

It must be noted that in the simulations reported here, the calculation of persistence and exchange times of both POPC and cholesterol near dimer interfaces is limited by the relative motion of one protomer with respect to one another. Thus, for these specific calculations, we used the results of previous simulations [14] of μ-OR crystallographic dimers, i.e., TM1,2,H8/TM1,2,H8 and TM5,6/TM5,6, where the relative orientation and distance of the protomers in the dimer were maintained constant. As illustrated in Fig. 7, the protein surface adjacent to the TM1,2,H8/TM1,2,H8 and TM5,6/TM5,6 dimer interfaces is in contact with regions of the membrane with slower dynamics. These regions of long persistence lipids right at the dimer interface may provide a mechanistic explanation for the preferential arrangement of receptors into extended linear arrays rather than compact or branched aggregates.

In summary, the simulations reported here suggest that both the formation of specific dimer interfaces and the overall topology of oligomeric aggregates depend on the kinetic k_on_ rates. Through calculation of both persistence and exchange times of lipid and cholesterol molecules during simulation, we show the presence of ‘jammed’ lipid regions that exhibit long persistence times and non-Poissonian dynamics, and we speculate that these regions play an important role in modulating the kinetics of GPCR di-/oligomerization.

Slower diffusion at the interface between integral membrane proteins and the membrane has been extensively investigated, leading to the distinction between annular and “non-annular” lipid behavior (e.g, see [26,27]). Averaged measures of lipid diffusion have been reported (e.g., see [27]) as a function of the radial distance from the receptor, and have showed a generally slower lipid motion at small radial distances from the protein surface. Although informative, these measures fail to discriminate between the different behavior of lipids adjacent to different helices of the receptor, and are therefore of limited use. While reported measurements of the extent of local time-averaged lipid displacement (proportional to the local average velocity of lipid molecules) [26] have allowed to identify regions of the protein surface likely to be in contact with slower lipids, the average velocity is not a direct measure of the membrane viscosity, thus complicating the interpretation of the results. The present analysis provides rigorous information about the local behavior of the viscosity, thus allowing to analyze its relation to OR dimer formation at specific interfaces.

The results reported here complement those of previous studies, and suggest the use of persistence and exchange times to assess the important role of metastable lipid structures in GPCR dimer formation. Our assessment of the rheological properties of the lipid bilayer also complements the analysis of membrane elasticity and mechanical properties via macroscopic empirical models [36–38]. In particular, the results reported here provide direct evidence for the effect of the microscopic dynamics of lipids and cholesterol at dimeric interfaces on the mesoscopic length- and time-scales of receptor interactions, further supporting an essential role of the lipid membrane in determining the identity of homomeric or heteromeric complexes of GPCRs.

## Materials and Methods

### System Preparation

The atomic coordinates of non-protein molecules were removed from the PDB files of the crystallographic structures of the mouse δ-OR (PDB ID: 4EJ4 [15]), mouse μ-OR (PDB ID: 4DKL [12]), and chain A of the human κ-OR (PDB ID: 4DJH [13]) receptors. Missing or unresolved residues (specifically, residues 263–270 in μ-OR and residues 262 and 301–307 for κ-OR) were built using ROSETTA [39]. The conformation for the intracellular loop 3 (IL3) was selected as the minimum energy structure reported by ROSETTA. For μ-OR, the lowest energy conformation that also did not interfere (inter-lock) with the IL3 of the adjacent receptor at the TM5/TM6 interface was selected. For δ-OR, the crystallographic IL3 was rebuilt using ROSETTA between residues 241 and 258. Notably, the root mean square deviation (RMSD) of this loop conformation from the resolved loop of the newest high-resolution crystal structure of δ-OR [40] is 0.46 Å overall. Throughout this article we use the Ballesteros-Weinstein numbering scheme to facilitate comparison between the receptor subtype TM regions [41]. Accordingly, the first number of this scheme indicates the TM helix in which the residue in question resides, and the second number indicates the position of the residue relative to the most conserved residue in the helix, which is always numbered 50. The receptors were converted to a CG representation under the MARTINI force field (version 2.1) [16–18] and a modified elastic network was applied, as reported previously in the literature [42]. According to recent experimental findings [35], the receptors were also palmitoylated at the C3.55 position, with a palmitoylate chain consisting of 4 C1 beads, with a bond length of 0.47 nm, a force constant of 1250 kJ mol-1 nm-2 and angles of 180°, with a force constant 25 kJ mol^−1^.

### Molecular Dynamics Simulations

Eighteen orientations (each rotated 20 degrees with respect to each other, thus covering 360 degrees around the z-axis of the receptor) of the CG receptors were each embedded in explicit CG POPC/10% cholesterol membranes (with a protein/lipid ratio of approximately 1:100) solvated with MARTINI CG water and neutralizing counterions. Temperature coupling at 300 K was achieved with the V-rescale algorithm, and pressure coupling at 1 bar was achieved with the Berendsen algorithm. Simulation were performed with GROMACS version 4.5.3 [43]. The systems were minimized and equilibrated with harmonic restraints of decreasing strength over 10 ns, on protein backbone beads. Water and counter ions were removed and sixteen of these membrane/protomer systems were randomly selected (repeat selections were permitted) and combined to create five 16-receptor setups for each of the studied homomeric (μ-OR/μ-OR, δ-OR/δ-OR, and κ-OR/κ-OR) and heteromeric (δ-OR/κ-OR and μ-OR/δ-OR) systems, which were re-solvated, neutralized, and subsequently minimized. The heteromeric systems contained eight receptors of each subtype. Thus, twenty-five independently constructed 16-receptor systems, each with approximately 57,000 beads, were generated. Each of these systems was simulated for 10 μs with a timestep of 20 fs, to give 50 μs of pooled simulation time for each homo- and heteromeric receptor system, and 250 microseconds in total, across all systems. Periodic boundary conditions were employed, and neighbor lists were updated every 10 steps. The Shift algorithm was used for electrostatic interactions. A single cut-off of 1.2 nm was used for Van der Waals interactions.

### Interface Identification and Clustering

For each receptor, we calculated the C_α_ contact map δ with all possible dimerization partners, and defined as “dimeric” the pairs for which at least 10 residues on each receptor were at a distance below an assigned cutoff (8 Å). Dimeric interfaces were clustered by a k-means algorithm using the distance induced by the Frobenius norm on the contact map matrices, and the clusters automatically labeled according to the TM regions involved in receptor-receptor interactions. Specifically, the dissimilarity of two interfaces k_1_ and k_2_ was defined as

$$d_{k_{1}k_{2}}=\left\|\delta_{ij}^{k_{1}}-\delta_{ij}^{k_{2}}\right\|$$

for hetero-dimers, while for homo-dimers the symmetrized form

$$d_{k_{1}k_{2}}=\min\left(\left\|\delta_{ij}^{k_{1}}-\delta_{ij}^{k_{2}}\right\|,\left\|\delta_{ij}^{k_{1}}-\delta_{ji}^{k_{2}}\right\|\right)$$

was used to account for the equivalence of the dimer with swapped protomers. The marginal contact map averages over the N_C_ interfaces *k* in cluster C were calculated for one receptor as

$$\delta_{j}^{\left(C\right)}=\frac{1}{N_{C}}\sum_{k\in C}\sum_{i}\delta_{ij}^{\left(k\right)}$$

while for the interacting one the analogous expression with *i* and *j* swapped inside the sum was used. Helices for which at least 3 residues were involved in the interface, were included in the label of the cluster.

We used a Bayesian inference framework to pool the information from the different trajectories and calculate estimates of the interface prevalence for each dimer and on-rates. The number of dimerization instances in a given trajectory *i* that yielded an interface belonging to cluster C can be described by a stochastic variable N_i,C_ with a multinomial distribution

$$p(N_{iC})=\frac{\sum_{C}N_{iC}}{\prod_{C}N_{iC}!}\prod_{C}p_{iC}^{N_{iC}}$$

where the probability of each cluster is p_i,C_ = w_C_/Σ_C_w_C_, w_C_ being the overall weight of the cluster. We defined w_C_ = exp(a_C_), used non-informative normal priors (with zero mean and large standard deviation) for the parameters a_C_, and employed Gibbs sampling to obtain the posterior distributions of p_C_, for which we report the average as well as (2.5%,97.5%) confidence intervals. While best estimates of the probabilities p_C_ are well approximated by the pooled fractions, the larger confidence intervals obtained by Bayesian inference reflect the variations in the different trajectories.

Estimates of the on-rate for dimerization k_on_(C) at each interface C were obtained assuming that associations are independent of each other, and that the number of interface-specific dimerization events *n* follows a Poisson distribution:

$$p(n)=\frac{{\left(t\;k_{\text{on}}(C)\;c_{i}\right)}^{n}}{n!}e^{-t\;k_{\text{on}}(C)\;c_{i}}$$

where *t* is the time and *c*_*i*_ the concentration of monomers in trajectory *i*. Again, we defined the parameters *b*_*Ci*_ so that the Poisson intensity is (t k_on_ c_i_) = exp(b_Ci_), where *b*_*Ci*_ have non-informative normal priors, and sampled the posterior distribution of *k*_on_ using Gibbs sampling. For all estimates, 10^4^ samples from the posterior distributions were obtained after a 5×10^3^ burn-in phase using Markov-chain Monte Carlo techniques [44].

Comparisons with available crystal structures of parallel interacting GPCRs (see S2 Table for a current list) were evaluated by calculating the overall Cα RMSD. In order to ignore the structural differences in the monomeric structures, and capture only the degree of similarity of the OR dimer interfaces from simulation with those inferred by crystal structures, we aligned the individual CG ORs to the receptors in each crystal dimer. For the evaluation of the identified hetero-dimeric interfaces, both pair combinations (e.g., μ-OR/δ-OR and δ-OR/μ-OR) were considered for the superposition onto crystal structures, and the RMSD was defined as the minimum between the two individual RMSD values.

### Lipid Velocity Distribution and Residence Times

We characterized the dynamical properties of the lipid (POPC) and cholesterol molecules by calculating their exchange and persistence time distributions (t_X_ and t_P_, respectively) at different positions relative to the protein. Specifically, we applied the equations reported in [25], and calculated local averages of lipid exchange and persistence time distributions, 〈t_X_〉, and 〈t_P_〉, respectively.

The persistence time is defined as the time it takes for a lipid molecule to move beyond a given cutoff distance *d* from its position at time 0, i.e. the minimum time for which ||x(t_P_)-x(0)||>*d*, (see S1, S2 Figs, and Figs. 5–6). Formally, this approach consists of viewing the lipid dynamics as a continuous time random walk (CTRW) model [45], defined by a coarse-graining length *d*, i.e. as a sequence of displacements of length *d* occurring at different times. The reported results for *d* = 10 Å do not change significantly for other choices of *d*. Increased persistence times t_P_ reflect long-lived microscopic arrangements of lipid molecules, thus representing a measure of structural correlation of the lipid motion or the membrane local viscosity ηα〈t_P_〉. The exchange time is the time elapsing between subsequent displacements of length *d*. Thus, the first exchange time t_x,1_ is the minimum time for which ||x(t_x,1_)-x(t_P_)||>d, where x is the position of the lipid, and the following values of the exchange time t_i_ are defined similarly by the condition ||x(t_x,i_)-x(t_x,(i-1)_)||>d.

In short, the persistence time t_P_ is the time the first step of the random walk occurs independent of the choice of the reference t = 0 time, whereas the exchange time t_X_ is the waiting time between two consecutive jump events. The latter characterizes the dynamics of the lipid molecules by a diffusion constant D∝1/〈t_X_〉. The distributions of persistence and exchange times, p_P_ and p_X_, respectively, are related by:

$$p_{P}(t)=\frac{1}{\langle t_{X}\rangle }\int_{t}^{\infty }ds\;p_{X}(s)$$

so that in normal bulk conditions, when p_X_ is Poissonian, so is p_P_, and 〈t_X_〉~〈t_P_〉.

In regions of locally restricted motion (“jammed” regions), such as crevices at the protein surface and regions between two protomers, jump events are clustered in time, typical persistence times can become larger than the exchange times, and 〈t_P_〉 is increased with respect to 〈t_X_〉, so that ηD ∝ 〈t_P_〉/〈t_X_〉 is no longer constant. The effective viscosity η increases and is decoupled from the diffusional motion of the lipids. We calculated the distributions p_P_ and p_X_ in bins of 1Å×1Å using the dynamics of the phosphate group in the first third of each trajectory, and averaging over upper and lower leaflet lipids, over all homomeric trajectories for a given receptor subtype, and over all protomers in the simulation, after alignment to a common reference protein molecule. Although the heteromeric simulations yielded comparable results, these are not reported here because of their reduced statistical significance.

### Position-Dependent Diffusion Coefficients

Position-dependent diffusion coefficients for all μ-OR protomers were calculated from the five 10 μs-long unbiased simulations carried out to simulate receptor homo-dimerization, using the Bayesian inference approach described previously in [31]. Continuous trajectories of pairs of interacting proteins were extracted from the original trajectories containing 16 copies of the receptor. To avoid problems with the interpretation of the results, frames in which a given interacting pair of protomers was in contact with a third protomer were discarded. This was achieved by checking that the distance between protomers in a given pair was smaller than distances from any other nearby protomer (see S3 Fig.). The time dependence of the two angles defining the relative orientation of the protomers (ϕ_1_, ϕ_2_) and their distance (*d*) was calculated for all the frames of the resulting trajectories of protomer pairs. Trajectories relative to the formation of specific interfaces were selected by restricting the analysis to regions I = {α_1_≤ϕ_1_≤β_1_ α_2_≤ϕ_2_≤β_2_}, and the distances {3 nm ≤ d ≤ 9 nm} binned to obtain a set of unbiased binned trajectories X = {d_j_(τ)}. The diffusion coefficient D was calculated by sampling the posterior distribution of the rate matrix from the posterior distribution p(K|X) = p(X|K) p(K), assuming a uniform prior p(K), and the likelihood

$$\ln p(X|K)={\sum_{jj^{\prime }}N_{jj^{\prime }}\ln\left(e^{\tau K}\right)}_{jj^{\prime }}$$

where N_jj’_ is the number of observed transitions between distance bin j and bin j’. From the distribution of the rate matrix K, the distribution of the diffusion coefficients was obtained from

$$D_{j}≃{\left(\Delta d\right)}^{2}K_{j,j+1}{\left(\frac{P_{j}}{P_{j+1}}\right)}^{1/2}$$

Average values of these quantities and their (25%, 95%) credible intervals are reported in S5 Table.

### Free-Energy Barrier Estimation from Steered MD

The free-energy barrier separating the μ-OR TM5/TM5 interface from simulations and the crystallographic TM5,6/TM5,6 interface was estimated using the Jarzynski equality [46] and multiple runs in which the angles ϕ_1_ = ϕ_2_ were steered from 1.2 degrees (corresponding to the TM5/TM5 interface) to 2.1 degrees (corresponding to the TM5,6/TM5,6 interface), with a velocity of 0.05 rad/ns and an elastic force of k = 800 (kcal mol^−1^)/rad^2^. The Jarzynski expression was applied separately to 5 sets of 5 independent runs to obtain the potential of mean force (PMF) and error estimates.

## Supporting Information

S1 Fig Exchange times for the lipids, in ns, around isolated δ-OR (panel A), κ-OR (panel B), and μ-OR (panel C). Approximate locations of the center of mass of the seven transmembrane (TM1-TM7) helices are indicated with red labels.
(TIF)

S2 Fig Exchange times for cholesterol molecules, in ns, around isolated δ-OR (panel A), κ-OR (panel B), and μ-OR (panel C). Approximate locations of the center of mass of the seven transmembrane (TM1-TM7) helices are indicated with red labels.
(TIF)

S3 Fig Coordinate system used in the calculation of the position-dependent diffusion coefficients of μ-OR protomers. Only frames in which no nearby protomers occupy the gray shaded region are included in the analysis. The relative position of the protomers is defined by the COM-COM distance *d*, and the angles of the COM distance with respect to the direction of the TM4 on each protomer.
(TIF)

S4 Fig Estimation of the free-energy difference between μ-OR dimer interfaces involving TM5. PMF of the transition from the observed TM5/TM5 interface (ϕ_1_ = ϕ_2_~1.2) and the crystallographic TM5,6/TM5,6 interface (ϕ_1_ = ϕ_2_~2.4) of μ-OR homo-dimers. Solid line is the average PMF obtained from the application of the Jarzynski equation to 5 independent sets of 5 simulations, and errors are the corresponding standard deviations.
(TIF)

S1 Table Summary of all the molecular dynamics simulations of coarse-grained representations of opioid receptors reported in this manuscript. (DOCX)

S2 Table Putative dimer interfaces inferred from recent GPCR crystal structures. (DOCX)

S3 Table Minimum RMSD distances (below 10 Å) between homo-dimeric complexes formed during simulation and selected crystal structures. For each cluster of dimeric complexes that formed during simulation, the configuration corresponding to the lowest RMSD (highlighted in bold) is depicted in Figs. 2, 3, and 4 of the manuscript for δ-OR/δ-OR, κ-OR/κ-OR, and μ-OR/μ-OR, respectively.
(DOCX)

S4 Table Minimum RMSD distances (below 10 Å) between hetero-dimeric complexes formed during simulation and selected crystal structures. (DOCX)

S5 Table Position-dependent diffusion rates calculated for the identified homo-dimeric configurations of μ-OR from simulations. Protein translational diffusion coefficients (in 10^−7^ cm^2^/s) for different interfaces and different distance ranges.
(DOCX)

S1 Movie Example of a simulation run for the homomeric δ-OR/δ-OR system. The instantaneous velocity of lipid molecules was calculated after interpolating the motion of the corresponding head groups over 20 ns. The density of lipids with velocity within the first quartile was calculated using a Gaussian kernel (with bandwidth 5 Å) and plotted in colors (blue, high density; white low density). The location of protein molecules is indicated approximately by plotting one single isodensity contour of the protein beads obtained with the same Gaussian kernel. The location of the center of mass of helices TM1, 4 and 6 is indicated with white, gray, and black dots, respectively.
(MP4)

S2 Movie Example of a simulation run for the homomeric κ-OR/κ-OR system. The instantaneous velocity of lipid molecules was calculated after interpolating the motion of the corresponding head groups over 20 ns. The density of lipids with velocity within the first quartile was calculated using a Gaussian kernel (with bandwidth 5 Å) and plotted in colors (blue, high density; white low density). The location of protein molecules is indicated approximately by plotting one single isodensity contour of the protein beads obtained with the same Gaussian kernel. The location of the center of mass of helices TM1, 4 and 6 is indicated with white, gray, and black dots, respectively.
(MP4)

S3 Movie Example of a simulation run for the homomeric μ-OR/μ-OR system. The instantaneous velocity of lipid molecules was calculated after interpolating the motion of the corresponding head groups over 20 ns. The density of lipids with velocity within the first quartile was calculated using a Gaussian kernel (with bandwidth 5 Å) and plotted in colors (blue, high density; white low density). The location of protein molecules is indicated approximately by plotting one single isodensity contour of the protein beads obtained with the same Gaussian kernel. The location of the center of mass of helices TM1, 4 and 6 is indicated with white, gray, and black dots, respectively.
(MP4)

S4 Movie Example of a simulation run for the heteromeric δ-OR/κ-OR system. The instantaneous velocity of lipid molecules was calculated after interpolating the motion of the corresponding head groups over 20 ns. The density of lipids with velocity within the first quartile was calculated using a Gaussian kernel (with bandwidth 5 Å) and plotted in colors (blue, high density; white low density). The location of protein molecules (red for δ-OR and black for κ-OR) is indicated approximately by plotting one single isodensity contour of the protein beads obtained with the same Gaussian kernel. The location of the center of mass of helices TM1, 4 and 6 is indicated with white, gray, and black dots, respectively.
(MP4)

S5 Movie Example of a simulation run for the heteromeric δ-OR/μ-OR system. The instantaneous velocity of lipid molecules was calculated after interpolating the motion of the corresponding head groups over 20 ns. The density of lipids with velocity within the first quartile was calculated using a Gaussian kernel (with bandwidth 5 Å) and plotted in colors (blue, high density; white low density). The location of protein molecules (red for δ-OR and black for μ-OR) is indicated approximately by plotting one single isodensity contour of the protein beads obtained with the same Gaussian kernel. The location of the center of mass of helices TM1, 4 and 6 is indicated with white, gray, and black dots, respectively.
(MP4)
