# Supplementary figures and images for: Preferred Supramolecular Organization and Dimer Interfaces of Opioid Receptors from Simulated Self-Association
Source: PLoS Comput Biol. 2015 Mar 30;11(3):e1004148. doi: 10.1371/journal.pcbi.1004148 (PMC4379167; doi:10.1371/journal.pcbi.1004148)

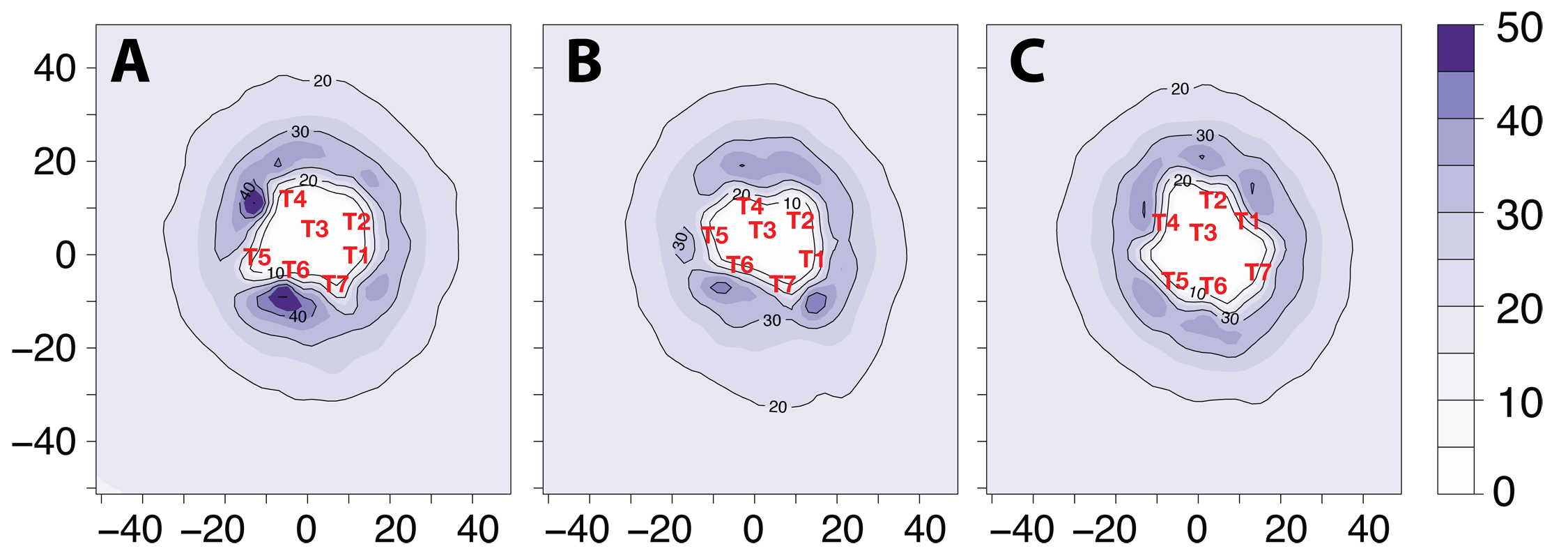

Supplement: S1 Fig — Approximate locations of the center of mass of the seven transmembrane (TM1-TM7) helices are indicated with red labels. (TIF) [file pcbi.1004148.s001.tif]

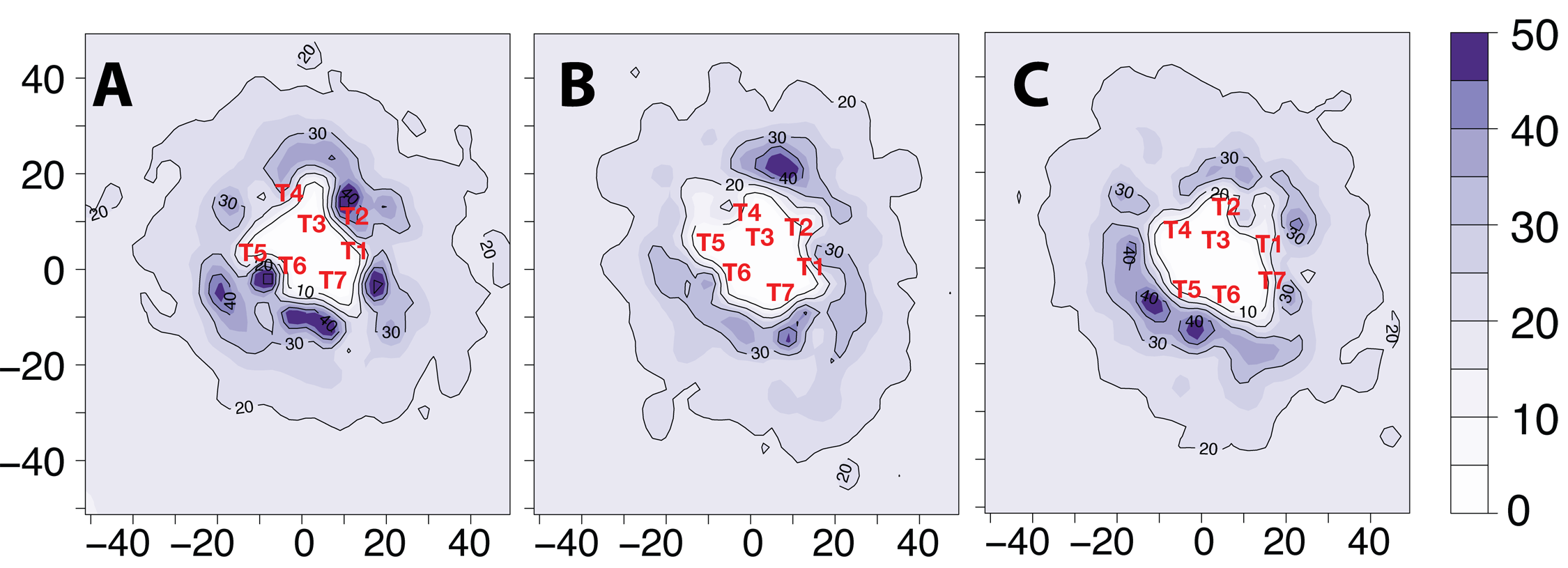

Supplement: S2 Fig — Approximate locations of the center of mass of the seven transmembrane (TM1-TM7) helices are indicated with red labels. (TIF) [file pcbi.1004148.s002.tif]

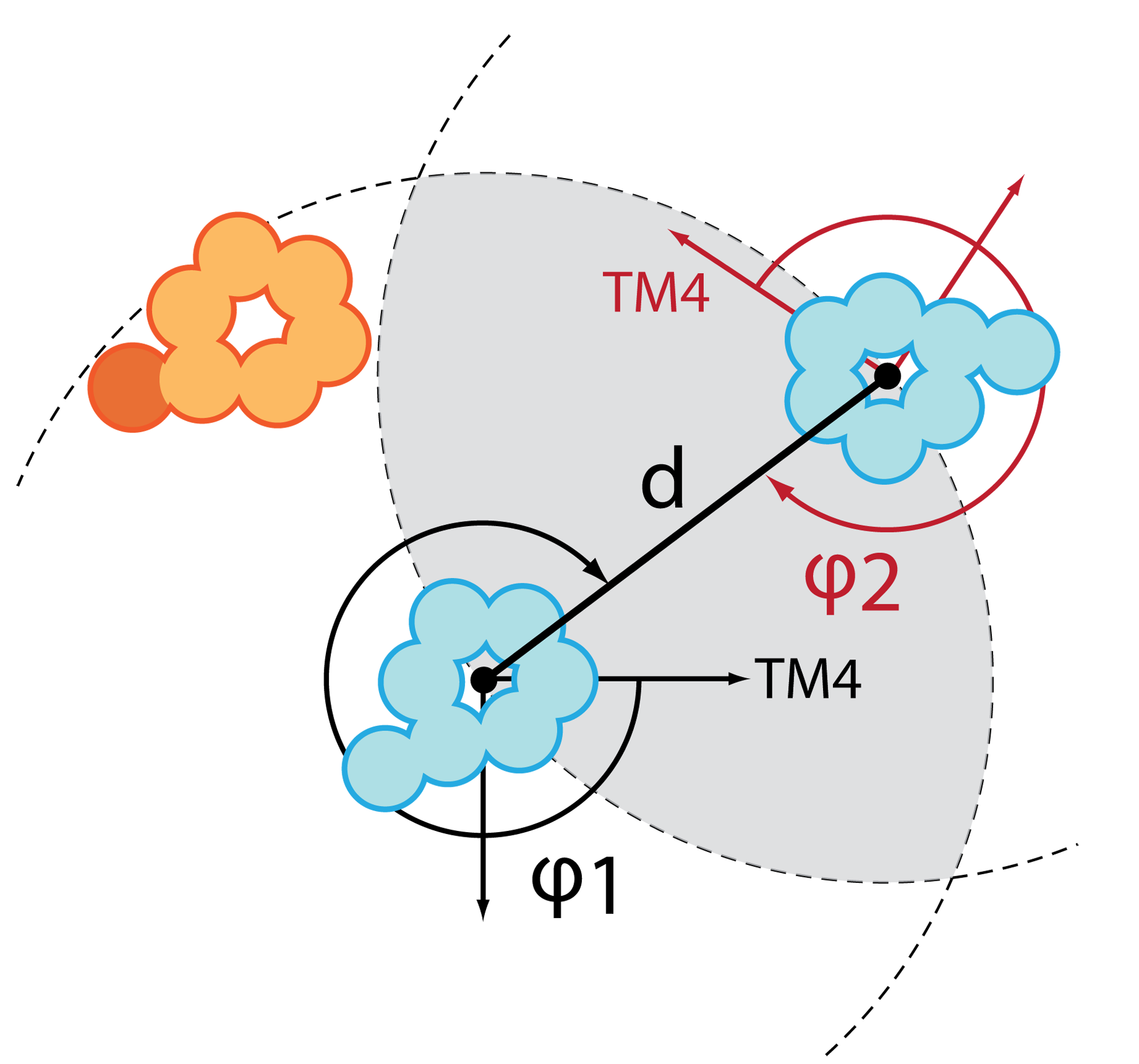

Supplement: S3 Fig — Only frames in which no nearby protomers occupy the gray shaded region are included in the analysis. The relative position of the protomers is defined by the COM-COM distance d, and the angles of the COM distance with respect to the direction of the TM4 on each protomer. (TIF) [file pcbi.1004148.s003.tif]

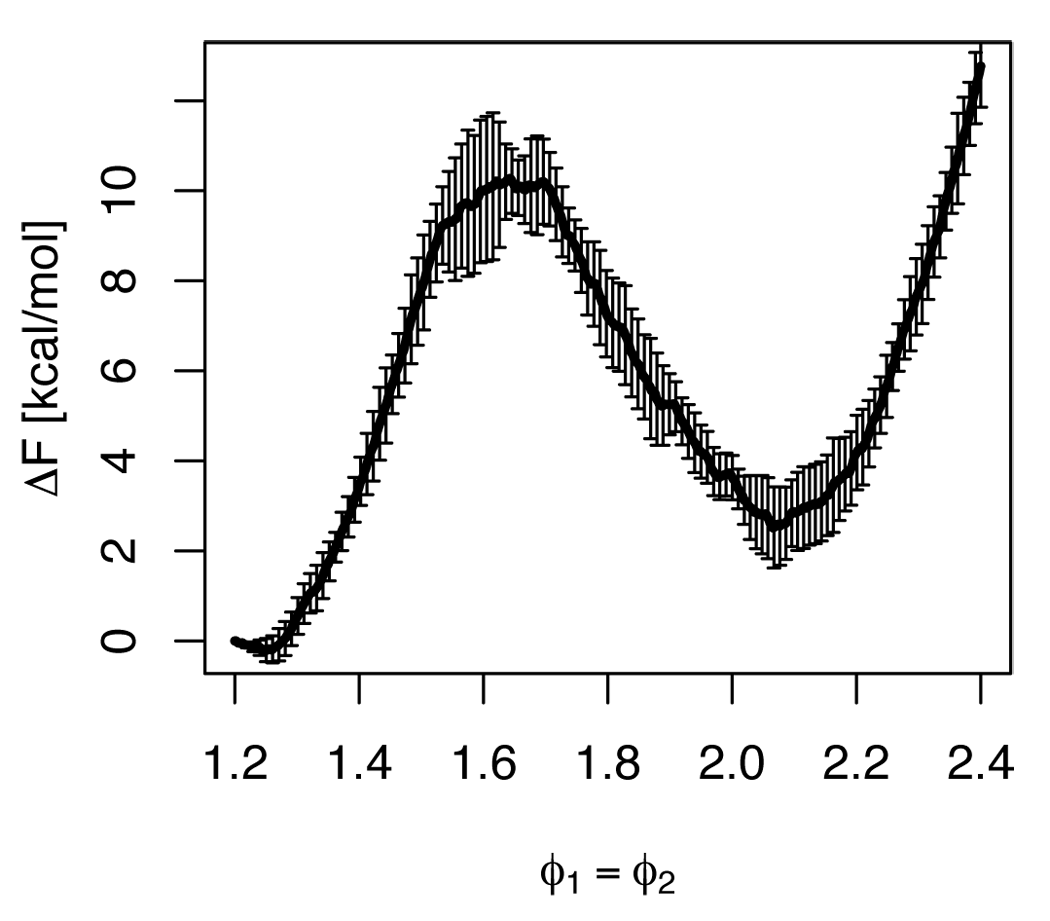

Supplement: S4 Fig — PMF of the transition from the observed TM5/TM5 interface (ϕ1 = ϕ2~1.2) and the crystallographic TM5,6/TM5,6 interface (ϕ1 = ϕ2~2.4) of μ-OR homo-dimers. Solid line is the average PMF obtained from the application of the Jarzynski equation to 5 independent sets of 5 simulations, and errors are the corresponding standard deviations. (TIF) [file pcbi.1004148.s004.tif]
