# Supplementary material for: Preferred Supramolecular Organization and Dimer Interfaces of Opioid Receptors from Simulated Self-Association
Source: PLoS Comput Biol. 2015 Mar 30;11(3):e1004148. doi: 10.1371/journal.pcbi.1004148 (PMC4379167; doi:10.1371/journal.pcbi.1004148)
Supplement: S1 Table — (DOCX) [file pcbi.1004148.s005.docx]

Table S1.

| **System** | **Total number of receptors** | **Number of runs** | **Simulation length (μs)** | **Total simulation time (μs)** |
| --- | --- | --- | --- | --- |
| **Homomeric** |  |  |  |  |
| δ-OR/δ-OR | 16 | 5 | 10 | 50 |
| μ-OR/μ-OR | 16 | 5 | 10 | 50 |
| κ-OR/κ-OR | 16 | 5 | 10 | 50 |
| **Heteromeric** |  |  |  |  |
| δ-OR/μ-OR | 8+8 | 5 | 10 | 50 |
| δ-OR/κ-OR | 8+8 | 5 | 10 | 50 |
