# Supplementary material for: Preferred Supramolecular Organization and Dimer Interfaces of Opioid Receptors from Simulated Self-Association
Source: PLoS Comput Biol. 2015 Mar 30;11(3):e1004148. doi: 10.1371/journal.pcbi.1004148 (PMC4379167; doi:10.1371/journal.pcbi.1004148)
Supplement: S2 Table — (DOCX) [file pcbi.1004148.s006.docx]

Table S2.

| **Receptor** | **PDB code** | **Reference** | **Dimer description** |
| --- | --- | --- | --- |
| opsin/rhodopsin | 3CAP (in representation of a number of rhodopsin structures, i.e. 2I36, 2I37, 2I35, and 4A4M, which show similar interfaces). | Park, J.H. et al., Nature (2008); 454(7201): 183-7. | TM1,H8/TM1,H8 (chains A and B in unit cell) |
| β1-adrenergic receptor (B1AR) | 4GPO | Huang, J. et al., Nat. Struct. Mol. Biol. (2013); 20(4):419-25. | TM1,2,H8/TM1,2,H8 (chains A and B in unit cell,), and TM4,5/TM4,5 (chain A and periodic image of chain B) |
| β2-adrenergic receptor (B2AR) | 2RH1 | Cherezov, V. et al., Science (2007); 318(5854): 1258-65. | TM1,H8/TM1,H8 (chain A with symmetry mate); N.B.: Interaction is mediated by lipids found at the interface. |
| chemokine receptor CXCR4 | 3ODU (in representation of other CXCR4 structures, i.e., 3OE9 and 3OE0, which show similar interfaces). | Wu, B, et al., Science (2010); 330(6007): 1066-71. | TM5,6/TM5,6 (chains A and B in unit cell; spacegroup P21); N.B. This interface is mostly mediated by the extracellular regions of TM5, with marginal contributions from the extracellular side of TM6, and additional contacts provided by the intracellular regions of TM3 and TM5.  . |
| chemokine receptor CXCR4 | 3OE8 | Wu, B, et al., Science (2010); 330(6007): 1066-71. | TM5,6/TM5,6 (chains B and C in unit cell; spacegroup P1) and TM1,2,H8/TM5,6,7 (chains A and B in unit cell). |
| chemokine receptor CCR5 | 4MBS | Tan, Q, et al., Science. (2013); 341(6152):1387-90. | TM1,2,H8/TM4 (chains A and B in unit cell). |
| κ-opioid receptor (κ-OR) | 4DJH | Wu, H. et al., Nature (2012); 485(7398): 327-32. | TM1,2,H8/TM1,2,H8 (chains A and B in unit cell). |
| μ-opioid receptor (μ-OR) | 4DKL | Manglik, A. et al., Nature (2012); 485(7398): 321-6. | TM5,6/TM5,6 (chain A with symmetry mate) and TM1,2,H8/TM1,2,H8 (chain A with symmetry mate). |
| smoothened (SMO) receptor | 4JKV | Wang, C. et al., Nature (2013); 497(7449): 338-43. | TM4,5/TM4,5 (chains A and B in unit cell). |
| metabotropic glutamate (mGlu1) receptor | 4OR2 | Wu et al, Science. 2014 Apr 4;344(6179):58-64. | TM1,2/TM1,2 (chains A and B in unit cell). |
