# Supplementary material for: Preferred Supramolecular Organization and Dimer Interfaces of Opioid Receptors from Simulated Self-Association
Source: PLoS Comput Biol. 2015 Mar 30;11(3):e1004148. doi: 10.1371/journal.pcbi.1004148 (PMC4379167; doi:10.1371/journal.pcbi.1004148)
Supplement: S3 Table — For each cluster of dimeric complexes that formed during simulation, the configuration corresponding to the lowest RMSD (highlighted in bold) is depicted in Figs. 2, 3, and 4 of the manuscript for δ-OR/δ-OR, κ-OR/κ-OR, and μ-OR/μ-OR, respectively. (DOCX) [file pcbi.1004148.s007.docx]

Table S3.

| **Interface** | **δ-OR/δ-OR** | **κ-OR/κ-OR** | **μ-OR/μ-OR** |
| --- | --- | --- | --- |
| TM1,2,H8/TM1,2,H8 | **3.57** Å **(4DJH)**  4.59 Å (4DKL)  7.81 Å (4GPO) | **4.13** Å **(4DJH)**  7.60 Å (4DKL)  5.34 Å (4GPO) | 6.73 Å (4DJH)  8.69 Å (4DKL)  **5.92** Å **(**4GPO**)** |
| TM1,2/TM4,5 | - | - | - |
| TM1,2/TM5,6 | **6.48** Å **(3OE8)** |  |  |
| TM4,5/TM4,5 | - | - | - |
| TM4,5/TM5,6 | 6.66 Å (3ODU)  **6.62 Å (3OE8)** | - | - |
| TM5/TM5 | - | **8.56** Å **(4DKL)** | 9.63 Å (3ODU)  **8.82** Å **(4GPO)** |
