# Supplementary material for: Preferred Supramolecular Organization and Dimer Interfaces of Opioid Receptors from Simulated Self-Association
Source: PLoS Comput Biol. 2015 Mar 30;11(3):e1004148. doi: 10.1371/journal.pcbi.1004148 (PMC4379167; doi:10.1371/journal.pcbi.1004148)
Supplement: S4 Table — (DOCX) [file pcbi.1004148.s008.docx]

Table S4.

| **Interface** | **δ-OR/κ-OR** | **δ-OR/μ-OR** |
| --- | --- | --- |
| TM1,2,H8/TM1,2,H8 | **4.23** Å **(4DJH)**  5.75 Å (4DKL)  8.07 Å (4GPO) | **4.22** Å **(4DJH)**  5.49 Å (4DKL)  9.11 Å (4GPO) |
| TM1,2/TM4,5 | - | - |
| TM1,2/TM5,6 | **7.15** Å **(3OE8)** | **8.60** Å **(3OE8)** |
| TM4,5/TM1,2 | - | - |
| TM4,5/TM5,6 | **7.27** Å **(3ODU)**  8.11 Å (**3OE8**) | - |
| TM5/TM1,2 | - |  |
| TM5/TM5 | - | **9.35** Å **(4DKL)** |
| TM5,6/TM1,2 | - | - |
