# Supplementary material for: Preferred Supramolecular Organization and Dimer Interfaces of Opioid Receptors from Simulated Self-Association
Source: PLoS Comput Biol. 2015 Mar 30;11(3):e1004148. doi: 10.1371/journal.pcbi.1004148 (PMC4379167; doi:10.1371/journal.pcbi.1004148)
Supplement: S5 Table — Protein translational diffusion coefficients (in 10−7 cm2/s) for different interfaces and different distance ranges. (DOCX) [file pcbi.1004148.s009.docx]

Table S5.

| **Interface** | **k_on_** (μm^2^/s) | **D_P_** (10^-7^ cm^2^/s) | | | |
| --- | --- | --- | --- | --- | --- |
|  |  | d<40Å | | 40Å ≤d≤50Å | d>50Å |
| TM1,2,H8/TM1,2,H8 | 4.0 | 0.6 (0.2,1.1) | | 1.3 (1.0,3.3) | 5.1 (4.1,7.3) |
| TM1,2/TM4,5 | 5.1 | 0.5 (0.1,2.2) | | 2.6 (1.2,3.4) | 5.3 (3.9,8.1) |
| TM1,2/TM5,6 | 19.4 | 2.1 (1.0,3.5) | | 1.7 (0.8,3.3) | 4.5 (3.5,7.0) |
| TM4,5/TM5,6 | 1.0 | 0.3 (0.2,0.7) | 2.1 (1.4,4.8) | | 3.3 (3.1,5.3) |
| TM5/TM5 | 22.7 | 2.5 (1.3,2.9) | 2.3 (1.2,4.3) | | 5.2 (4.4,5.8) |
